# Alzheimer’s disease is associated with prostate cancer: a population-based study

**DOI:** 10.18632/oncotarget.24139

**Published:** 2018-01-10

**Authors:** Herng-Ching Lin, Li-Ting Kao, Shiu-Dong Chung, Chung-Chien Huang, Ben-Chang Shia, Chao-Yuan Huang

**Affiliations:** ^1^ School of Health Care Administration, Taipei Medical University, Taipei, Taiwan; ^2^ Sleep Research Center, Taipei Medical University Hospital, Taipei, Taiwan; ^3^ Graduate Institute of Life Science, National Defense Medical Center, Taipei, Taiwan; ^4^ Division of Urology, Department of Surgery, Far Eastern Memorial Hospital, Banciao, New Taipei City, Taiwan; ^5^ Big Data Research Center, Taipei Medical University, Taipei, Taiwan; ^6^ Department of Urology, National Taiwan University Hospital, College of Medicine National Taiwan University, Taipei, Taiwan; ^7^ School of Public Health, Taipei Medical University Hospital, Taipei, Taiwan

**Keywords:** Alzheimer’s disease, prostate cancer, epidemiology

## Abstract

Alzheimer’s disease and cancer are increasingly prevalent with advancing age. However, the association between Alzheimer’s disease and prostate cancer remains unclear. The aim of this study was to examine the relationship between prior Alzheimer’s disease and subsequent prostate cancer using a population-based dataset in Taiwan. Data for this study were sourced from the Taiwan Longitudinal Health Insurance Database 2005. This case-control study included 2101 prostate cancer patients as cases and 6303 matched controls. We used conditional logistic regression analyses to calculate the odds ratio (OR) and corresponding 95% confidence interval (CI) for Alzheimer’s disease between prostate cancer patients and controls. We found that of the 8404 sampled patients, 128 (1.5%) had been diagnosed with Alzheimer’s disease prior to the index date. A Chi-squared test showed that there was a significant difference in the prevalences of prior Alzheimer’s disease between prostate cancer patients and controls (2.1% vs. 1.3%, *p* < 0.001). The conditional logistic regression analysis showed that the OR of prior Alzheimer’s disease for prostate cancer patients was 1.53 (95% CI: 1.06∼2.21) compared to controls. Furthermore, the OR of prior Alzheimer’s disease for prostate cancer patients was 1.52 (95% CI: 1.04∼2.22) compared to controls after adjusting for hypertension, diabetes, coronary heart disease, hyperlipidemia, obesity, prostatitis, gonorrhea or chlamydia infection, testitis or epididymitis, and alcohol abuse/alcohol dependency syndrome. This study revealed an association between prior Alzheimer’s disease and prostate cancer. We suggest that clinicians be alert to the increased risk of prostate cancer when caring for elderly individuals with Alzheimer’s disease.

## INTRODUCTION

Alzheimer’s disease (AD), the fifth leading cause of death in adults over 65 years of age in the United States, is one of the most prevalent neurodegenerative disorders worldwide [1]. In the United States, an estimated 5.2 million individuals over 65 years of age had AD in 2012 [2], and the number of people who develop AD is expected to increase by almost triple, to 13.5 million by 2050 [3, 4]. AD and cancer are increasingly prevalent with advancing age, and the probability of the co-occurrence of both AD and cancer in the same patient also rises with increasing age [5].

Prostate cancer (PC) is the second major cause of cancer-related mortality among older males in Western countries, and androgen deprivation therapy (ADT) is the first-line treatment for patients with PC [6, 7]. However, only two prior retrospective cohort studies explored the association between dementia and PC [4, 8], but those studies showed contradictory results. In addition, some studies further examined the association between AD or dementia and the use of ADT for treating PC [9–13]. However, their findings on the association between the use of ADT and dementia were inconsistent, and some methodological limitations were observed in those studies, such as small sample sizes, absence of consideration of exposure lag periods, and short follow-up periods. Furthermore, the majority of the above studies focused on the association between PC and the subsequent risk of dementia, and very few studies attempted to investigate the association between prior dementia or AD and PC. The lack of such studies prevents researchers from understanding the underlying mechanisms between these two medical conditions.

Therefore, the aim of this study was to examine the relationship between prior AD and subsequent PC using a population-based dataset in Taiwan.

## RESULTS

We found that of the 8404 sampled patients, the mean age was 74.1 ± 9.5 (± standard deviation) years. After matching for age, monthly income, geographical location, urbanization level of the patient’s residence, and index date, Table 1 shows that there were significant differences in hyperlipidemia (*p* < 0.001), obesity (*p* = 0.008), prostatitis (*p* < 0.001), and testitis or epididymitis (*p* = 0.043) between PC patients and controls.

**Table 1:** Demographic characteristics of patients with prostate cancer and controls in Taiwan (*n* = 8404)

Variable	Patients with prostate cancer (*N* = 2101)		Controls (*N* = 6303)		*p* value
	Total no.	Percent (%)	Total no.	Percent (%)	
Age, mean (SD), years	74.1 (9.5)		74.1 (9.5)		>0.999
Urbanization level					>0.999
1 (most urbanized)	640	30.5	1920	30.5	
2	607	28.9	1821	28.9	
3	291	23.8	873	23.8	
4	313	14.9	939	14.9	
5 (least urbanized)	250	11.9	750	11.9	
Monthly income					>0.999
≤NT15,840	1212	57.7	3636	57.7	
NT$15,841∼25,000	601	28.6	1803	28.6	
≥NT$25,001	288	13.7	864	13.7	
Geographic region					>0.999
Northern	1059	50.4	3177	50.4	
Central	497	23.7	1491	23.7	
Southern	497	23.7	1491	23.7	
Eastern	48	2.2	144	2.2	
Obesity	21	1.0	30	0.5	0.008
Hypertension	1547	73.6	4677	74.2	0.605
Diabetes mellitus	675	32.1	2073	32.9	0.519
Testitis or epididymitis	47	2.2	99	1.6	0.043
Gonorrhea or chlamydia infection	31	1.5	67	1.1	0.127
Hyperlipidemia	945	45.0	2527	40.1	<0.001
Alcohol abuse/alcohol dependence syndrome	4	0.2	20	0.3	0.345
Prostatitis	295	14.0	333	5.3	<0.001

The average exchange rate in 2014 was US$1.00≈New Taiwan (NT)$29.

Table 2 shows the prevalences of prior AD between PC patients and controls. Of the 8404 sampled patients, 128 (1.5%) had been diagnosed with AD prior to the index date. A Chi-squared test showed that there was a significant difference in the prevalences of prior AD between PC patients and controls (2.1% vs. 1.3%, *p* < 0.001).

**Table 2:** Prevalence for prior Alzheimer’s disease among sampled subjects

Presence of prior Alzheimer’s disease	Total (*N* = 8404)		Patients with prostate cancer (*N* = 2101)		Controls (*n* = 6303)	
	*n*, Percent (%)		*n*, Percent (%)		*n*, Percent (%)	
Yes	128	1.5	43	2.1	85	1.3
No	8276	94.5	2058	97.9	6218	98.7

Notes: There was a significant association between prostate cancer and prior Alzheimer’s disease (*p* < 0.001).

The OR and its corresponding 95% CI for having been previously diagnosed with AD between PC patients and controls are presented in Table 3. The conditional logistic regression (conditioned on age, monthly income, geographical location, urbanization level of the patient’s residence, and index date) showed that the OR of prior AD for PC patients was 1.53 (95% CI: 1.06∼2.21) compared to controls. Furthermore, the OR of prior AD for PC patients was 1.52 (95% CI: 1.04∼2.22) compared to controls after adjusting for hypertension, diabetes, coronary heart disease, hyperlipidemia, obesity, prostatitis, gonorrhea or chlamydia infection, testitis or epididymitis, and alcohol abuse/alcohol dependency syndrome. We also found that PC was positively and significantly associated with obesity (adjusted OR = 1.77, 95% CI = 1.00∼3.14), hyperlipidemia (adjusted OR = 1.21, 95% CI = 1.09∼1.35), and prostatitis (adjusted OR = 2.79, 95% CI = 2.36∼3.30). However, PC was negatively and significantly associated with diabetes (adjusted OR = 0.88, 95% CI = 0.79∼0.99).

**Table 3:** Crude and adjusted odds ratios (ORs) of prostate cancer among sampled patients

Variable	Prostate cancer	
	Crude OR (95% CI)	Adjusted OR (95% CI)
Prior Alzheimer’s disease	1.53^*^ (1.06∼2.21)	1.52^*^ (1.04∼2.22)
Obesity	2.11^**^ (1.21∼3.70)	1.77^*^ (1.00∼3.14)
Hypertension	0.97 (0.87∼1.09)	0.96 (0.86∼1.09)
Diabetes mellitus	0.97 (0.87∼1.07)	0.88^*^ (0.79∼0.99)
Testitis or epididymitis	1.43^*^ (1.01∼2.04)	1.31 (0.91∼1.97)
Gonorrhea or chlamydia infection	1.39 (0.91∼2.14)	1.46 (0.95∼2.26)
Hyperlipidemia	1.22^***^ (1.11∼1.35)	1.21^***^ (1.09∼1.35)
Alcohol abuse/alcohol dependence syndrome	0.60 (0.21∼1.76)	0.58 (0.20∼1.73)
Prostatitis	2.93^***^ (2.48∼3.46)	2.79^***^ (2.36∼3.30)

Note: ^*^p < 0.05, ^**^p < 0.01, ^***^p < 0.001. The adjusted odds ratios were derived from a conditional logistic regression model and adjusted for all other variables. CI, confidence interval.

## DISCUSSION

This population-based case-control study found an association between prior AD and PC. To the best of our knowledge, this is the first study to report an association between AD and PC. We found that patients with PC were 1.53-times more likely than controls to have had a previous diagnosis of AD. Even after adjusting for medical comorbidities, patients with PC were still at 1.52-times greater risk than comparison subjects for having a diagnosis of prior AD.

Two previous studies reported on the association between dementia and PC [5, 8]. One retrospective cohort study by Raji *et al.* included 106,061 patients aged 68 years or older with breast, colon, or PC in the United State and evaluated the risks of mortality from cancer and non-cancer causes, stratified by the presence or absence of preexisting dementia [5]. They found that a dementia diagnosis was associated with increased odds of being diagnosed at an unknown stage of PC. Another retrospective cohort study by Lin *et al.* included 3282 subjects with dementia and investigated the risk of cancers occurring after a diagnosis of dementia [8]. Those authors found that the adjusted HR for PC among dementia patients was 0.44 (95% CI = 0.20∼0.98) during a 7-year follow-up period compared to controls. However, that study did not take potential confounders such as prostatitis, and gonorrhea or chlamydia infection into consideration in their study.

The association of AD and PC is supported by some plausible biologic mechanisms including through abnormal deposits of proteins [20, 21], neuronal cell death [5, 13, 22], and oxidative stress [23, 24]. First, the amyloid precursor protein (APP) is a type I transmembrane protein that produces various proteolytic products due to alternative splicing [25]. Among its products, β-amyloid is generated by sequential and abnormal cleavage of the APP in the central nervous system, and was implicated in the development of AD [26, 27]. Previous studies also showed that the APP is a primary androgen-responsive gene that promotes the growth of PC cells, and that its high immunoreactivity is connected with poor prognoses in patients with PC [27, 28]. Therefore, sequential and abnormal cleavage of the APP might be one potential explanation for the association between AD and PC.

The second plausible explanation might be neuronal cell death. Neuronal cell death is one of the pathognomonic neurofibrillary tangle formations in AD [13]. Graham *et al.* also showed that these protein posttranslational modifications result in destabilization of the cytoskeleton, which in turn results in observed neuronal cell death [22]. Moreover, cancer is characterized by uncontrolled cell proliferation [8]. Consequently, neuronal cell death caused by a malfunction in cell growth regulation might be one potential mechanism for the association between AD and PC. Moreover, oxidative stress was shown to play an important role in the pathophysiology of neuron degeneration and death in AD [29, 30]. In addition, induction of oxidative stress from ADT in PC patients could produce reactivation of androgen receptor signaling in a hormone-refractory manner [31, 32]. Thus, oxidative stress might be implicated in the association between AD and PC. Furthermore, the action of acetylcholinesterase inhibitors in patients with AD is considered to be a potential mechanism underlying AD-associated tumorigenesis. To date, the acetylcholinesterase inhibitors are the common managements for AD [33]. Nevertheless, increasing biological evidences indicated that acetylcholinesterase activity in cancer is decreased, which may further contribute to cancer cell proliferation [34, 35]. Therefore, downregulated acetylcholinesterase activity in AD patients receiving acetylcholinesterase inhibitors might be one potential elucidation for the relationship between AD and PC.

This study has a number of strengths. The use of a longitudinal population-based database can decrease selection bias and avoid recall bias which often occur in case-control studies. Moreover, over 98% of Taiwan’s residents are of Chinese Han ethnicity, so the homogenous population may exempt our study from potential confounding by race. Nevertheless, this study possesses limitations that warrant consideration. First, studies that rely on diagnostic codes for an AD diagnosis are susceptible to misclassification [36, 37]. Detailed medical records, including biochemical tests, Mini-Mental State Examination (MMSE) scores, and medical imaging were not available in the database. However, we only included those AD cases who had received prescriptions of AChEIs, which needs to be approved by a committee including neurologists or psychiatrists, in order to increase the accuracy of the clinical diagnosis of AD. Second, the database also provides no lifestyle information or laboratory records, including inflammatory biomarkers, family history, and genetic factors. These factors might affect cognitive function and impact the association between prior AD and subsequent PC [38, 39]. Third, most patients involved in this case-control study were of Chinese Han ethnicity. Therefore, the findings in this study might not be generalized to other ethnic groups with high PC prevalence. Last, the current study design does not permit an unequivocal inference of a causal relationship between AD and PC.

Despite these limitations, this population-based case-control study revealed an association between AD and PC. We suggest that clinicians be alert to the increased risk of PC when caring for elderly individuals with AD. Further large-scale epidemiological, experimental, or pathological studies are necessary to determine the mechanisms underlying this association between prior AD and subsequent PC.

## METHODS

### Database

The data for this study were from the Longitudinal Health Insurance Database 2005 (LHID2005). The LHID2005, which is derived from medical claims records of the Taiwan National Health Insurance (NHI) program, consists of medical claims and registration files for 1,000,000 enrollees under the Taiwan NHI program. The Taiwan National Health Institute randomly selected these 1,000,000 enrollees from all enrollees (25.68 million) listed in the 2005 Registry of Beneficiaries. The LHID2005, which is open to researchers in Taiwan, offers an excellent opportunity to clarify the relationship between AD and PC using a population-based study.

This study was exempt from full review by the Institutional Review Board of Taipei Medical University (TMU-JIRB N201612023) since the LHID2005 consists of de-identified secondary data released without restriction to researchers for research purposes.

### Study sample

This case-control study comprised cases (patients with PC) and matched controls. As for the selection of cases, 2154 patients with a first-time diagnosis of PC (ICD-9-CM code 185) during an ambulatory care visit between January 2007 and December 2013 were identified. We excluded 53 patients under 50 years of age because of a very low prevalence of PC and AD in this age group. As a result, 2101 PC patients were included as cases. We further assigned the first claim date with a diagnosis of PC as the index date for cases.

We selected male controls from the remaining male beneficiaries of the LHID2005. We initially assured that none of the selected controls had ever received a diagnosis of PC in any claim. We then selected three controls (*n* = 6303) per case matched by age, monthly income (NT$0∼15,840, NT$15,841∼25,000, ≥NT$25,001; the average exchange rate in 2008 was US$1.00≈New Taiwan (NT)$29), geographical location (northern, central, southern, and eastern), urbanization level of the patient’s residence, and year of the index date. The year of the index date for cases was the year in which they received the first claim date with a diagnosis of PC. For controls, the year of the index date for controls was the matched year in which the controls had at least one episode of utilization of ambulatory care. Furthermore, we defined the date of first utilization of ambulatory care occurring in the index year as the index date for controls.

### Exposure assessment

This study identified AD cases based on ICD-9-CM codes 290 and 331.0. In addition, we only included those AD cases who had received prescriptions of acetylcholinesterase inhibitors (AChEIs) in order to increase the validity of the AD diagnoses. In Taiwan, prescribing AChEIs for patients with AD needs to be approved by a committee including neurologists or psychiatrists. This committee evaluates whether those patients are entitled for reimbursement for AChEIs according to patients’ clinical records, cognitive function, biochemistry tests, and diagnostic imaging. Furthermore, we only included those AD cases who had received at least one AD diagnoses before the index date.

### Statistical analysis

The SAS system for Windows (vers. 8.2, SAS Institute, Cary, NC) was used to perform all statistical analyses in this study. Chi-squared tests were carried out to explore differences in sociodemographic characteristics, hypertension, diabetes, hyperlipidemia, prostatitis, gonorrhea or chlamydia infection, testitis or epididymitis, obesity, and alcohol abuse/alcohol dependence syndrome between PC patients and controls. In addition, this study used ICD-9-CM codes to identify those cases with obesity (ICD-9-CM codes 278) and alcohol abuse/alcohol dependence syndrome (ICD-9-CM codes 291.1, 291.2, 291.5, 291.8, 291.9, 303.90–303.93, 305.00–305.03, V113). We then used conditional logistic regression analyses (conditioned on age, monthly income, geographical location, urbanization level of the patient’s residence, and index date) to calculate the odds ratio (OR) and the corresponding 95% confidence interval (CI) for AD between PC patients and controls. Additionally, the medical comorbidities, such as hypertension, diabetes, hyperlipidemia, prostatitis, gonorrhea or chlamydia infection, testitis or epididymitis, obesity, and alcohol abuse/alcohol dependence syndrome, were considered in the adjustment models in this study, because they were all potential confounders that might affect the association between AD and PC [14–19]. We used the conventional *p* ≤ 0.05 to assess statistical significance.
